# Inappropriate mode switch and unwarranted right ventricular pacing during atrial fibrillation: Paradoxical atrial undersensing

**DOI:** 10.1002/joa3.12791

**Published:** 2022-10-19

**Authors:** Katsuhide Hayashi, Junjiroh Koyama, Ken Okumura, Haruhiko Abe, Bruce L. Wilkoff

**Affiliations:** ^1^ Cleveland Clinic Cleveland Ohio USA; ^2^ Saiseikai Kumamoto Hospital Kumamoto Japan; ^3^ University of Occupational and Environmental Health, Japan Kitakyushu Japan

**Keywords:** atrial fibrillation, electromagnetic interference, noise detection, paradoxical atrial undersensing, undersensing

A 83‐year‐old male with a history of catheter ablation therapy for paroxysmal atrial fibrillation 7 years ago had palpitations and presyncope for 2 months. Implantable loop recorder revealed 6 s sinus pauses correlated with symptoms of presyncope. A dual‐chamber pacemaker Edora 8 DR‐T® (Biotronik, Berlin, Germany), right atrial lead (Solia S45, Biotronik), and right ventricular lead (Solia S53, Biotronik) were implanted. The pacemaker was set to the DDD mode (DDIR as mode switch mode) with the lower and upper rate limits set to 60 and 120 ppm, and atrial sensitivity set to 0.5 mV. The measured P‐wave amplitude was 4.4–5.2 mV during sinus rhythm.

Four hours later after the procedure, the patient developed palpitations at rest and his ECG monitor recorded an intermittently fast and irregularly ventricular paced rhythm with rates up to the programmed upper rate limit of 120 bpm. Pacemaker interrogation documented atrial fibrillation (AF) with amplitudes of 0.5–3.0 mV. Atrial lead impedance was 507 Ω and about the same as those at the implantation. After a while of observation, the sensitivity was re‐programming to 0.2 mV from 0.5 mV since there were some failure to sense AF electrogram (Figure 1A). Subsequently, there was a delay in detecting the AF, mode switching was inconsistent and there was intermittent rapid tracking of AF with ventricular pacing (Figure 1B). Virtually all AF electrogram amplitudes were larger than 0.5 mV, but when the sensitivity was re‐programmed to 0.2 mV, the frequency of AF detection deteriorated and there was more rapid ventricular pacing (Figures 1 and 2). As a troubleshooting intervention, ultimately we programmed the pacemaker to the DDI mode since the atrial electrogram was not reliably interpreted by the device as AF. Ultimately the atrial sensitivity set to 0.5 mV and pilsicainide was initiated to prevent AF.

**FIGURE 1:**
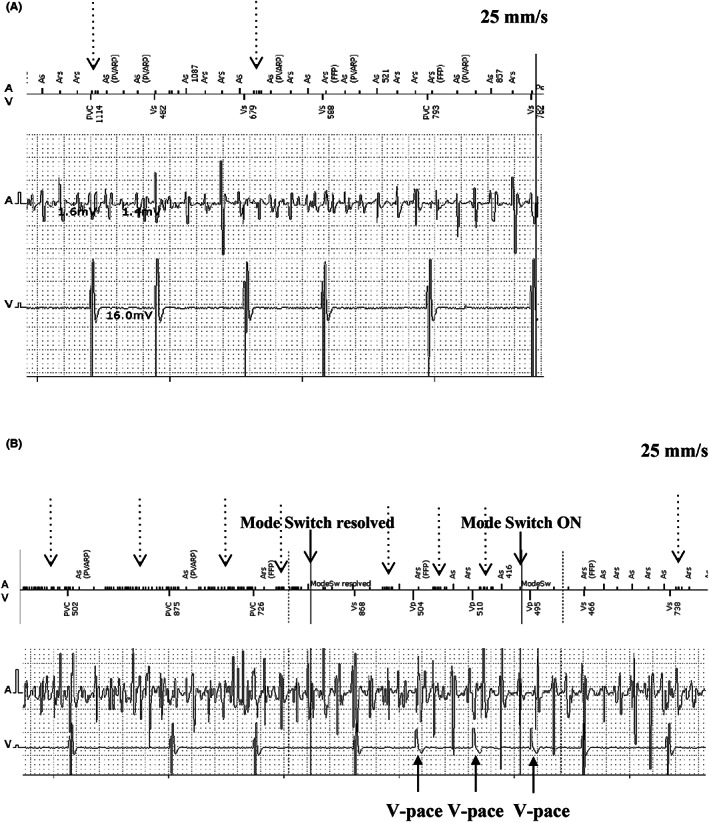
Intracardiac electrograms. Upper: Marker channel diagram, middle: Intracardiac atrial electrogram. Lower: Intracardiac ventricular electrogram. (A) Atrial sensitivity at 0.5 mV. The most atrial sensing function worked properly but there was some failure to sense AF electrogram (indicated by dotted arrow). (B) Atrial sensitivity 0.2 mV. There was failure to sense almost AF electrograms (indicated by dotted arrow) and mode switch was resolved by judging no atrial tachyarrhythmia event as a result. Thereafter some AF electrograms that were sensed made fast ventricular pacing tracked and mode switch were on again. As, atrial sensing; Ars, atrial refractory sensing; PVARP, postventricular refractory period; FFP, far‐field protection; PVC, premature ventricular contraction; Vs, ventricular sensing; Vp, ventricular pacing

**FIGURE 2:**
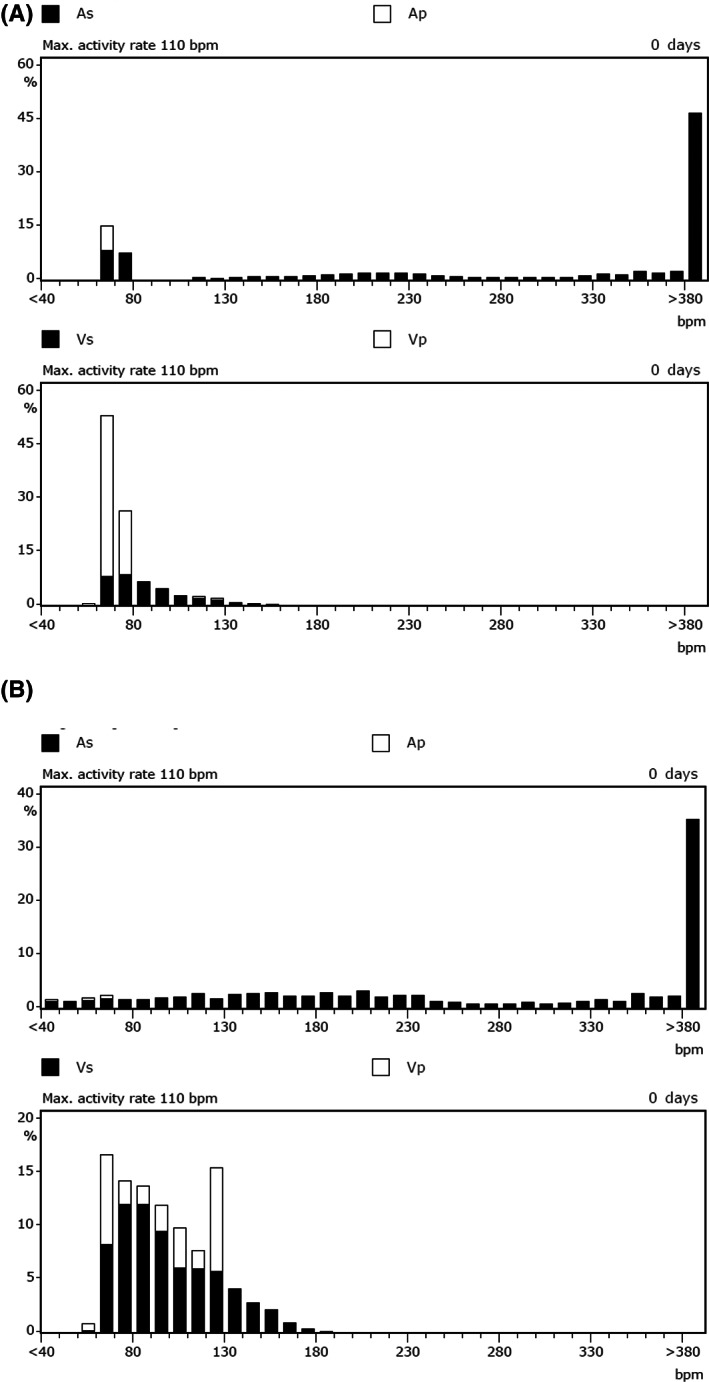
Rate histograms. (A) Atrial sensitivity 0.5 mV. Atrial rate histogram shows high rate sensing event indicating AF rhythm in most of atrial event in upper row. (B) Atrial sensitivity 0.2 mV. Atrial rate histogram shows high rate sensing event indicating AF rhythm in most of atrial event in upper row. However, ventricular rate histogram indicates frequent upper rate (120 bpm) pacing event. This indicates inappropriate ventricular tracking pace during AF. This phenomenon was getting worse by change from 0.5 mV to 0.2 mV at atrial sensitivity. As, atrial sensing; Ap, atrial pacing; Vs, ventricular sensing; Vp, ventricular pacing

Programming a low atrial sensing level (a high numerical value) may cause under detection of the atrial rhythm if the atrial signal amplitude is smaller than the sensitivity setting level. Therefore, to reduce the incidence of atrial undersensing, a high sensitivity setting is often chosen to assure detection of atrial arrhythmias as well as the normal atrial signal. However, increasing the sensitivity setting may also cause inappropriate detection of atrial arrhythmias in the setting of myopotentials or far‐field R‐wave sensing.^1^

Paradoxical atrial undersensing, undersensing as a result of increasing the atrial sensitivity has also been reported.^2^, ^3^, ^4^, ^5^ This is caused by the triggering of quiet timer blanking intervals after a large intrinsic atrial signal by the sensing amplifier as described below.

This case demonstrates intermittent undersensing during AF despite adequate electrogram amplitudes. As the atrial sensitivity was increased (programmed from 0.5 mV to 0.2 mV), the atrial undersensing became worse. This paradoxical response was reported by Willems et al. in an animal model.^2^ Pacemakers start a retriggerable atrial refractory period with every atrial sensed atrial event. When another atrial event is detected within this refractory period, the retriggerable period is reinitiated. If this continues for an interval longer than the lower rate interval, the pacemaker switches to the noise reversion mode, usually asynchronous pacing/VOO mode (Figure 3). When the pacemaker comes out of the noise mode, AF detection needs to start over and the AF electrograms are again tracked to the ventricle.

**FIGURE 3:**
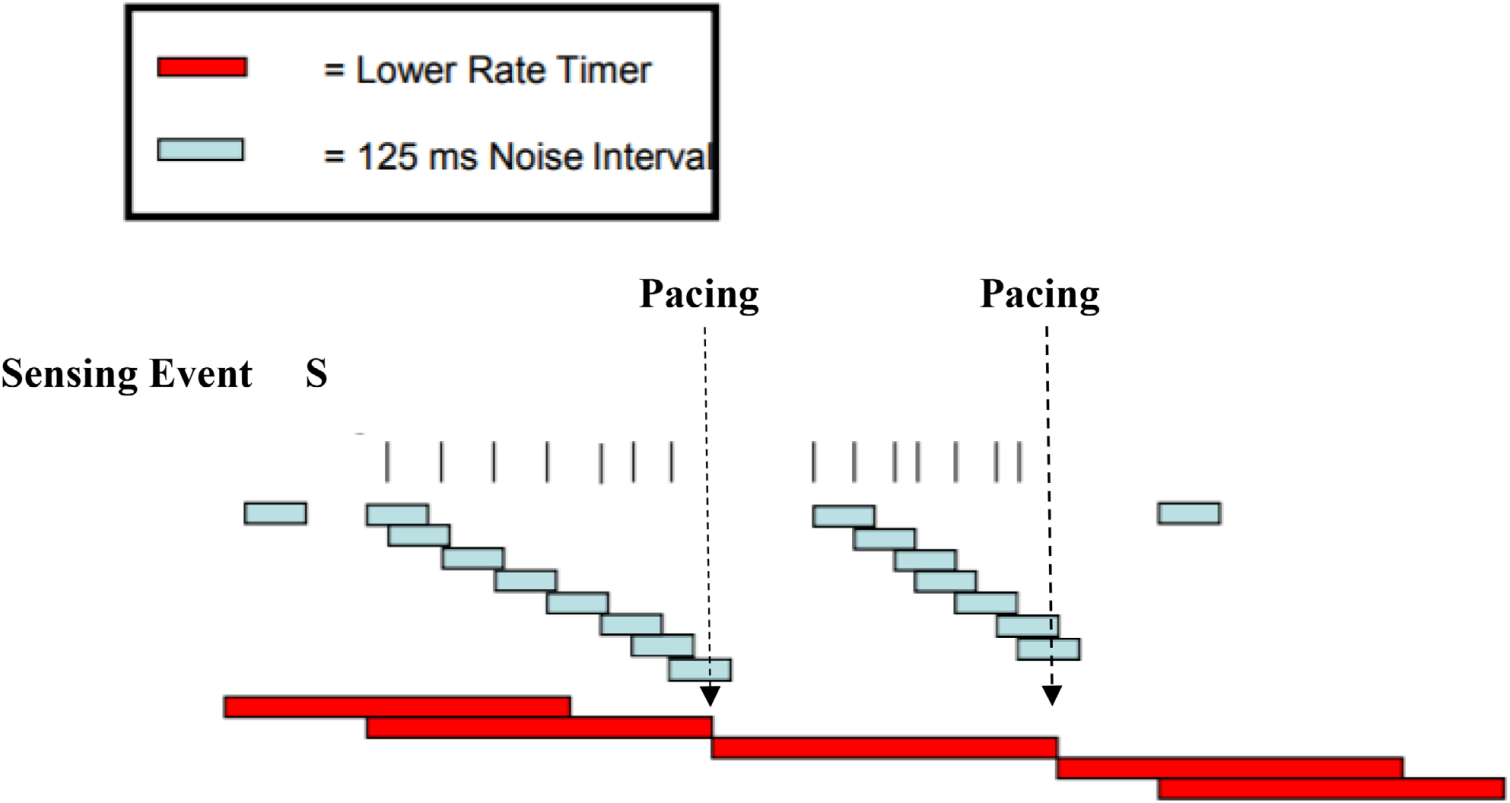
Noise mode pacing behavior in BIOTRONIK pacemakers (note: Example uses 125 ms initial noise interval). Sensed events within noise interval will trigger another noise interval. If sensing events within noise interval continues through the expiration of the lower rate interval, the pacemaker produces asynchronous pacing at a basic rate. (figure is used with permission from BIOTRONIK)

The setting method of retriggerable atrial refractory periods differs depending on each pacemaker model and manufacturer. Triggering of the noise mode behavior in BIOTRONIK pacemakers is shown in Figure 3. In Edora 8 DR‐T® made by Biotronik, noise time interval is non programmable and set at 100 ms. In the present case, the large amplitude and split or fragmented AF electrograms repetitively reset the noise time intervals by oversensing AF electrograms (Figure 3). This resulted in mode switch resolving by judging no atrial tachyarrhythmia. Thereafter some AF electrograms that were sensed made fast ventricular pacing tracked and mode switch were on again by rapid atrial sensing event.

The paradox can sometimes be avoided by decreasing the sensitivity level appropriately, such as ≥1 mV.^3^, ^4^ Morishima et al concluded that decreasing the sensitivity to the optimal value while confirming there is no atrial undersensing during sinus rhythm could be a solution to the problem of paradoxical atrial undersensing in their case report.^4^ In their case, the amplitudes of AF electrograms was 5.6–8.0 mV, therefore true undersensing of AF was unlikely to occur when the atrial sensitivity was set at 1.4 mV. However, in the present case, the atrial sensitivity was set at 0.5 mV to avoid true undersensing with 0.5–3.0 mV amplitudes during AF. As a troubleshooting intervention, we programmed from the DDD mode to DDI mode in order to not track the atrial activity, eliminating cycle length variability.

If undersensing of atrial fibrillation is suspected from the surface ECG, considering the possibility of paradoxical atrial undersensing is recommended before lead revision is considered. Present case is an example of infrequent but clinically relevant phenomenon that is a result of a feature of the pacemaker and not inherent atrial sensing that would be resolved with lead revision or lower sensitivity.

In conclusion, paradoxical atrial undersensing can be observed at high sensitivity level in patients with high amplitude of the AF. Knowledge of this phenomenon is important to avoid inappropriate Mode Switch and unwarranted RV pacing during AF and atrial lead revisions.

## CONFLICT OF INTEREST

Katsuhide Hayashi, Junjiroh Koyama, Haruhiko Abe; None. Ken Okumura; Honoraria: Medtronic. Bruce L. Wilkoff; Consultant and Honoraria: Abbott, Boston Scientific, Medtronic, Biotronic, Philips, Convatec, Xcardia.
